# Single-dose Ceftriaxone Plus 7-Day Doxycycline Versus Single-dose Benzathine Penicillin G Plus 7-Day Doxycycline in the Treatment of Early Syphilis in People With HIV: a Pilot Randomized Noninferiority Trial

**DOI:** 10.1093/ofid/ofag483

**Published:** 2026-08-08

**Authors:** Tzong-Yow Wu, Kuan-Yin Lin, Hsin-Yun Sun, Yu-Shan Huang, Wang-Da Liu, Aristine Cheng, Wang-Huei Sheng, Wen-Chun Liu, Li-Hsin Su, Yi-Ching Su, Chien-Ching Hung

**Affiliations:** Department of Internal Medicine, National Taiwan University Hospital Yunlin Branch, Yunlin, Taiwan; Department of Internal Medicine, National Taiwan University Hospital and National Taiwan University College of Medicine, Taipei, Taiwan; Department of Internal Medicine, National Taiwan University Hospital and National Taiwan University College of Medicine, Taipei, Taiwan; Department of Internal Medicine, National Taiwan University Hospital and National Taiwan University College of Medicine, Taipei, Taiwan; Department of Internal Medicine, National Taiwan University Hospital and National Taiwan University College of Medicine, Taipei, Taiwan; Department of Internal Medicine, National Taiwan University Hospital and National Taiwan University College of Medicine, Taipei, Taiwan; Department of Internal Medicine, National Taiwan University Hospital and National Taiwan University College of Medicine, Taipei, Taiwan; Department of Internal Medicine, National Taiwan University Hospital and National Taiwan University College of Medicine, Taipei, Taiwan; Department of Internal Medicine, National Taiwan University Hospital and National Taiwan University College of Medicine, Taipei, Taiwan; Department of Internal Medicine, National Taiwan University Hospital and National Taiwan University College of Medicine, Taipei, Taiwan; Department of Internal Medicine, National Taiwan University Hospital Yunlin Branch, Yunlin, Taiwan; Department of Internal Medicine, National Taiwan University Hospital and National Taiwan University College of Medicine, Taipei, Taiwan; Department of Tropical Medicine and Parasitology, National Taiwan University College of Medicine, Taipei, Taiwan

**Keywords:** chlamydia, gonorrhea, sexually transmitted infection, *Treponema pallidum*

## Abstract

**Background:**

Both ceftriaxone (CRO) and doxycycline demonstrate activity against *Treponema pallidum* and bacteria causing sexually transmitted infections (STIs); however, the efficacy of their combination in a shorter duration remains unclear. We compared single-dose, 1-g CRO plus 7-day doxycycline (CRO/Doxy) with single-dose, 2.4-MU benzathine penicillin G plus 7-day doxycycline (BPG/Doxy) in treating early syphilis among people with HIV (PWH).

**Methods:**

In this pilot randomized clinical trial, eligible participants were randomized to receive CRO/Doxy or BPG/Doxy. Rapid plasma reagin (RPR) titers were determined at baseline and at weeks 4, 12, 24, 36 and 48. PCR assays were conducted at baseline and at week 4 to detect *T. pallidum* and other STI-causing bacteria in oral rinse, urethral swab, and rectal swab samples to assess microbiologic responses. The primary outcome was the serologic responses of syphilis, defined as a 4-fold or greater decline of RPR titer at week 24.

**Results:**

From March 2023 to September 2024, 56 and 53 PWH with early syphilis were randomized to receive CRO/Doxy and BPG/Doxy, respectively. At baseline, 31.2% (n = 34) had chlamydia, 13.8% (n = 15) gonorrhea, and 6.4% (n = 7) *Mycoplasma genitalium* coinfection. In the ITT analysis, compared to the participants receiving BPG/Doxy, those receiving CRO/Doxy showed a lower serologic response rate at 24 weeks (60.7% vs 75.5%; difference, −14.8 percentage points; 95% CI, −32.0 to 2.5). The microbiologic response rates of syphilis, chlamydia, and *M. genitalium* infection were comparable between groups.

**Conclusions:**

In the ITT analysis, CRO/Doxy was not noninferior to BPG/Doxy for early syphilis treatment among PWH at 24 weeks.

## Teaser

Treatment with a single dose of ceftriaxone plus a 7-day course of doxycycline did not demonstrate non-inferiority compared to that with a single dose of BPG plus a 7-day course of doxycycline among PWH with early syphilis at 24 weeks.

## Graphical Abstract

**Graphical Abstract:**
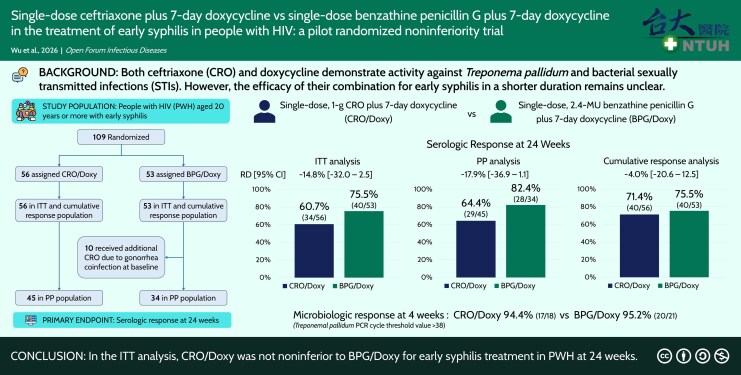

In recent years, global incidence of syphilis has been rising, partly due to shifts in sexual behaviors and the widespread implementation of pre-exposure prophylaxis (PrEP) for human immunodeficiency virus type 1 (HIV-1) [1]. Benzathine penicillin G (BPG) remains the first-line therapy for early syphilis [2]. However, the current management of syphilis poses several challenges. First, co-infection with other STIs, such as *Chlamydia trachomatis* and *Neisseria gonorrhoeae* infection, is not uncommon in PWH with early syphilis [3]. These co-infections necessitate additional treatment other than BPG and require these individuals to attend a second clinic visit, a step that may be challenging. Second, global shortages of BPG in recent years have posed significant challenges in clinical care [4]. Third, although penicillin desensitization is an established approach for individuals with penicillin allergy, it may not always be successful [5]. Finally, the global resistance of *Treponema pallidum* to azithromycin has increased, and evidence supporting the use of other antibiotics remains limited [6].

Ceftriaxone (CRO) is a recognized alternative therapy for early syphilis [2]. It exhibits a terminal half-life of 8 hours [7, 8]. Historical *in vitro* studies showed that CRO achieves 50% treponemal immobilization at a minimum concentration of 0.01 μg/mL [9]. A recent study using a continuous *in vitro* culture system demonstrated growth inhibition of *T. pallidum* at CRO concentrations of 0.0025 μg/mL [10]. Pharmacokinetic data reported that a single, 1.0-g dose of CRO results in a serum concentration at 24 hours of 29.7 μg/mL [11]. These data suggest that a single dose of CRO may maintain serum concentrations above the level required to inhibit *T. pallidum* growth for several days. Additionally, CRO has superior cerebrospinal fluid (CSF) penetration compared to BPG, offering a potential advantage in treating asymptomatic neurosyphilis. Studies administering CRO over 7–21 days have shown it to be comparably effective to single-dose BPG for the treatment of early syphilis [12–16].

Several arguments may support a potential new combination therapy for early syphilis using CRO and doxycycline, with both agents given for shorter durations than the standard of care. First, given that a single, 1.0-g dose of CRO may maintain treponemicidal serum concentrations for an extended period, single-dose administration may be a practical option that is both effective and convenient, while also providing treatment for concomitant gonorrhea. Second, several studies demonstrated that combination therapy was associated with improved serologic response in individuals with early syphilis [17–19]. Third, some studies support the addition of a 7-day course of doxycycline to conventional treatment in PWH with early syphilis [18, 19]. One retrospective study reported higher serologic response rates with single-dose BPG plus CRO and doxycycline than with BPG alone [17]. Another study showed that a single-dose BPG combined with 7-day doxycycline (BPG/Doxy) improved the 12-month serologic response in PWH compared with BPG alone [18]. These findings support the rationale for combining single-dose CRO with a 7-day doxycycline regimen (CRO/Doxy) for individuals with early syphilis. However, the clinical efficacy with CRO combining Doxy for early syphilis has not been previously established.

This study aimed to compare the efficacy with CRO/Doxy versus BPG/Doxy for the treatment of early syphilis among people with HIV (PWH).

## METHODS

### Study Design and Participants

We conducted an open-label, single-center, non-inferiority, randomized clinical trial at the infectious diseases clinics at the National Taiwan University Hospital, Taiwan. PWH aged 20 years or more with early syphilis, that is, primary, secondary, and early latent syphilis, were recruited. The syphilis stage was classified in accordance with US Centers for Disease Control and Prevention classifications [2]. The diagnosis of early syphilis was confirmed by the combination of clinical symptoms and serologic tests. PWH were excluded if they had a rapid plasma reagin (RPR) titer of less than 4; exposure to antibiotics with activity against *T. pallidum* within the preceding 4 weeks, that is, penicillins, third-generation cephalosporins, Doxy, and macrolides; a known or suspected infection prior to enrollment that required additional treatment with an antimicrobial active against *T. pallidum*; a history of intolerance to penicillin, CRO, or Doxy; or pregnancy. Participants were randomized in a 1:1 ratio to receive either CRO/Doxy or BPG/Doxy. Randomization was conducted using a computer-generated assignment sequence and was stratified based on syphilis stage. The study was approved by the Research Ethics Committee of the hospital (registration number, NTUH-202206138MIND) and all participants provided written informed consent before enrollment. The trial was registered with ClinicalTrials.gov (NCT05980871).

### Procedures

At baseline, all participants were asked to complete a self-administered questionnaire interview, which assessed their socioeconomic status, symptoms suggestive of STIs, history of STIs, sexual behaviors, and substance use. Participants underwent physical examination and clinical assessment of syphilis. Laboratory assessments included serologic tests for syphilis (*T. pallidum* particle agglutination assay and non-treponemal assay), and self-collected oral rinse, rectal swab, and urethral swab samples for PCR identification of *T. pallidum, C. trachomatis*, *N. gonorrhoeae*, and *Mycoplasma genitalium* DNA using previously described methods [3]. Swab specimens were collected from non-lesion sites.

Participants assigned to the CRO/Doxy group received a single intramuscular dose of 1.0 g CRO combined with Doxy 100 mg twice daily for 7 days, while those to the BPG/Doxy group received a single intramuscular dose of 2.4 million units (MU) of BPG combined with Doxy 100 mg twice daily for 7 days. Both CRO and BPG were injected at the upper buttock area. All the participants were interviewed by telephone 1 week after initiating treatment to assess adverse events related to the medication and adherence. Subsequently, follow-up visits were scheduled at weeks 4, 12, 24, 36, and 48 (study end) and included a repeat interview and physical examination. RPR tests and PCR identification of *T. pallidum* were repeated at weeks 4, 12, 24, 36, and 48. PCR identification of *C. trachomatis*, *N. gonorrhoeae*, and *M. genitalium* was repeated at week 4.

### Laboratory Investigations

The serological tests of syphilis were performed using a reactive RPR test (BD Macro-Vue RPR Card tests) for screening, followed by confirmation using the *T. pallidum* particle agglutination assay (FTI-SERODIA-TPPA; Fujirebio Taiwan Inc., Taoyuan, Taiwan). CD4 cell count was determined using flow cytometry (BD FACS Calibur; Becton Dickinson, CA), and plasma HIV RNA load was quantified using the Cobas AmpliPrep/Cobas TaqMan HIV-1 test (version 2.0; Roche Molecular Systems, Pleasanton, CA) (lower detection limit, 20 copies/mL). An in-house PCR assay was used to amplify the 47 kDa antigen gene of *T. pallidum* from oral rinse, rectal swab, and urethral swab samples, following a method previously described [20]. A multiplex real-time PCR assay was used to identify *C. trachomatis*, *N. gonorrhoeae*, and *M. genitalium* from oral rinse, rectal swab, and urethral swab samples (Allplex STI Essential Assay; Seegene Inc, Seoul, Republic of Korea).

### Outcomes

The primary endpoint was serologic response at week 24, defined as either a 4-fold or greater decline in RPR titer compared to baseline or seroreversion to a nonreactive result. Secondary endpoints included the following: (1) microbiologic response of syphilis at week 4, defined as *T. pallidum* PCR cycle threshold (Ct) value >38 [20]; (2) microbiologic response of bacterial STIs at week 4, defined as negative PCR results; (3) serologic response of syphilis at weeks 12 and 48; and (4) adverse events, including injection site pain or local swelling, symptoms and signs suggestive of Jarisch-Herxheimer reaction such as fever, chills, skin flushing, and tachycardia within 24 hours after injection, and other adverse events, based on records from the telephone interview.

Participants who did not achieve clinical or serological response, or experienced recurrences, were defined as treatment failure, and were subsequently treated with a single dose of 2.4 MU of BPG at the discretion of the physician. This study did not perform genotypic analysis and, therefore, could not exclude cases of reinfection among individuals classified as treatment failure.

### Statistical Analysis

This study was conducted under the assumption that CRO/Doxy would be non-inferior to BPG/Doxy for the primary endpoint. Non-inferiority would be established if the lower limit of the 95% confidence interval (CI) for the between-group difference in serologic response rates did not exceed the prespecified non-inferiority margin of −10%. Based on a randomized trial by Cao et al. that compared CRO with BPG for the treatment of early syphilis, we assumed that 90% of the participants who received CRO and 78% of those who received BPG would achieve serologic responses at week 24. Under these assumptions, we initially calculated that at least 150 participants per treatment group would be required to detect a between-group difference with 80% power at a two-sided significance level of 5%. However, we identified an error in the original sample size calculation and determined that enrollment of 43 participants per group would have been sufficient to achieve 80% power to establish non-inferiority of CRO/Doxy [16]. Consequently, recruitment was terminated early. We used the intention-to-treat (ITT) population for the primary analysis, which included all randomized participants. Participants who did not achieve a ≥ 4-fold decline in RPR titers, subsequently received BPG, or missed follow-up were classified as treatment failures. Missing data were classified as treatment failures in the analysis. Two secondary analyses were also conducted. The first was a per-protocol (PP) analysis, which excluded participants with a baseline co-infection requiring additional antibiotics active against *T. pallidum*, those who received such antibiotics during follow-up, or those missed follow-up. Participants who received additional antibiotics with potential anti-treponemal activity were retained in the ITT analysis but excluded from the PP analysis. The second analysis was a cumulative-response analysis, which included all randomized participants; participants were considered to have permanently achieved the primary endpoint upon demonstrating a serologic response.

Categorical variables were compared using Fisher's exact test or Chi-square test, while non-categorical variables were compared using the Mann-Whitney U test. The time to serologic response was depicted by Kaplan-Meier curves and compared by log-rank tests. Parametric variables are compared using t-test or ANOVA. A logistic regression model was conducted to identify factors associated with serologic response. For multivariable analyzes, we included variables with a *P* value <.1 in univariable analyzes. The effects of each variable on serologic response were estimated with 95% confidence intervals (CIs) of odds ratios (ORs). All comparisons were two-tailed, and a *P* value <.05 was considered significant. Statistical analyses were performed with the use of STATA software version 18.0 (Stata Corporation, College Station, TX).

## RESULTS

**Figure 1.**
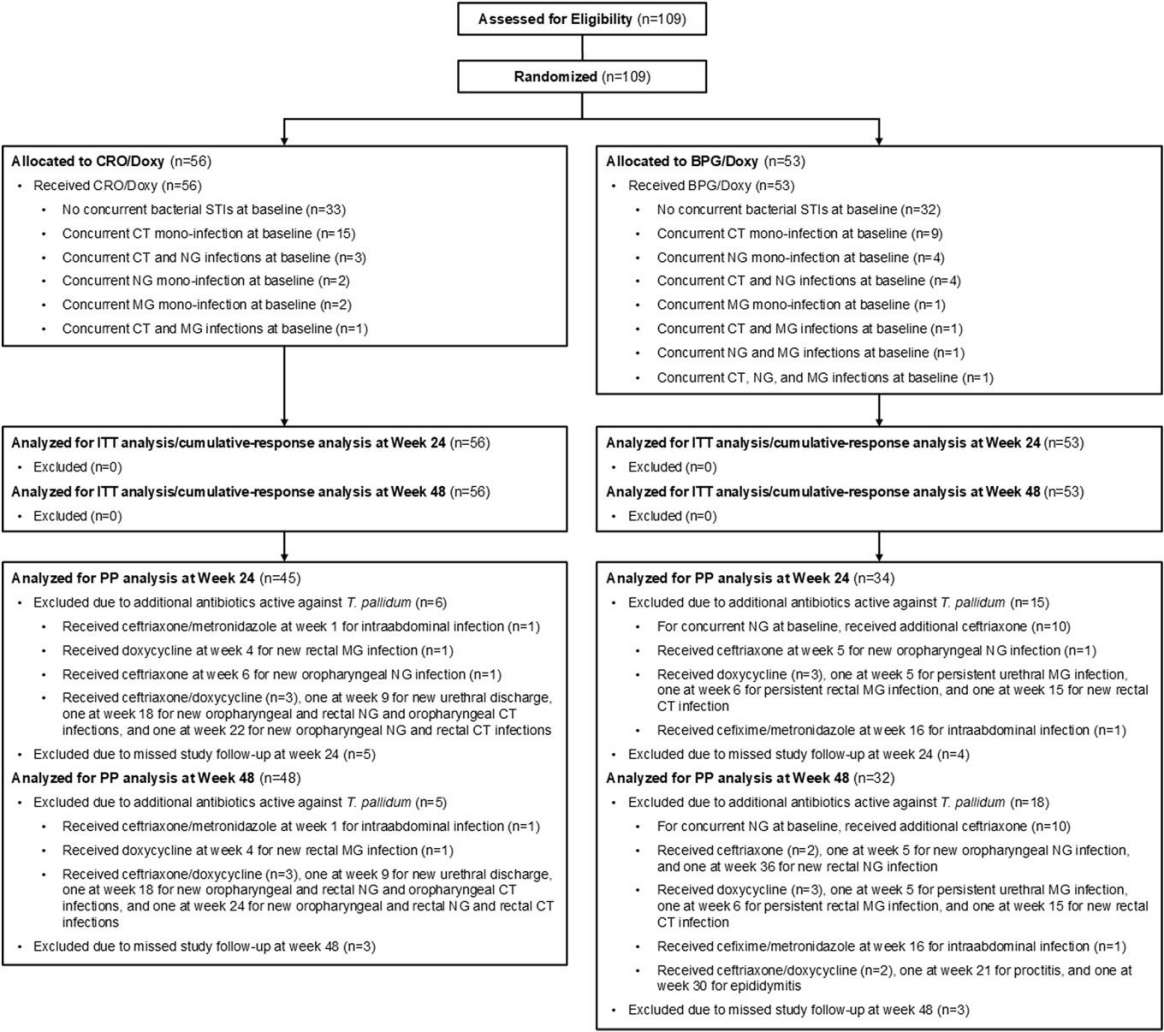
Flow diagram. To treat concurrent gonorrhea at baseline, 18.9% (10/53) of PWH in the BPG/Doxy group received additional ceftriaxone. At 48 wks, 8.9% (5/56) of PWH receiving CRO/Doxy and 34.0% (18/53) of PWH receiving BPG/Doxy had received additional antibiotics active against *T. pallidum* during follow-up. Abbreviations: ITT, intention-to-treat; PP, per-protocol; BPG, benzathine penicillin G; CRO, ceftriaxone; Doxy, doxycycline; CT, *Chlamydia trachomatis*; NG, *Neisseria gonorrhoeae*; MG, *Mycoplasma genitalium.*

Between 10 March, 2023, and 16 August, 2024, a total of 109 PWH with early syphilis were assessed for eligibility and randomized to receive either CRO/Doxy (n = 56) or BPG/Doxy (n = 53) (Figure 1). Ten participants (18.9%) in the BPG/Doxy group also received CRO because of detection of *N. gonorrhoeae* at baseline. Baseline demographic and clinical characteristics were similar between the two groups (Table 1). All participants were men who have sex with men (MSM), with a median age of 39 years (interquartile range [IQR], 34–46). The median CD4 count was 558 cells/mm^3^ (IQR, 449–774), and 87.2% (n = 95) had plasma HIV RNA levels <200 copies/mL. Most participants (85.3%, n = 93) had a prior history of syphilis. Seven (6.4%), 38 (34.9%) and 64 (58.7%) participants had primary, secondary and early latent syphilis, respectively. *T. pallidum* DNA was detectable in 35.8% (n = 39) of the 109 participants at enrollment. Co-infection rates were 31.2% (n = 34) for chlamydia, 13.8% (n = 15) for gonorrhea, and 6.4% (n = 7) for *M. genitalium* infection. All participants with co-infections were asymptomatic.

**Table 1.** Baseline Characteristics of Participants

Baseline Characteristics	Total (N = 109)	CRO/Doxy Group (N = 56)	BPG/Doxy Group (N = 53)
Male, n (%)	109 (100)	56 (100)	53 (100)
Age, median (IQR), y	39 (34–46)	38 (33–46)	40 (35–46)
Receiving ART at baseline, n (%)	108 (99.1)	56 (100)	52 (98.1)
CD4 count at baseline, median (IQR), cells/mm^3^	558 (449–774)	556 (477–794)	565 (446–733)
Plasma HIV viral load <200 copies/mL, n (%)	95 (87.2)	51 (91.1)	44 (83.0)
HBsAg positivity, n (%)	9 (8.3)	7 (12.5)	2 (3.8)
HCV infection^a^, n (%)	23 (21.1)	13 (23.2)	10 (18.9)
History of previous syphilis, n (%)	93 (85.3)	49 (87.5)	44 (83.0)
Syphilis stage, n (%)	**…**	**…**	**…**
Primary	7 (6.4)	3 (5.4)	4 (7.5)
Secondary	38 (34.9)	22 (39.3)	16 (30.2)
Early latent	64 (58.7)	31 (55.4)	33 (62.3)
RPR titer, median (IQR)	128 (64–256)	128 (64–256)	128 (64–256)
RPR titer ≤ 1:32, n (%)	24 (22.0)	12 (21.4)	12 (22.6)
RPR titer ≥ 1:64, n (%)	85 (78.0)	44 (78.6)	41 (77.4)
*T. pallidum* DNA detected^b^, n (%)	39 (35.8)	18 (32.1)	21 (39.6)
Oral site, n (%)	33/39 (84.6)	15/18 (83.3)	18/21 (85.7)
Rectal site, n (%)	17/39 (43.6)	8/18 (44.4)	9/21 (42.9)
Urethral site, n (%)	10/39 (25.6)	2/18 (11.1)	8/21 (38.1)
≥2 sites, n (%)	16/39 (41.0)	7/18 (38.9)	9/21 (42.9)
Interval between RPR testing to treatment administration, median (IQR), days	5 (1–9)	5 (0–9)	5 (1–9)
Concurrent bacterial STIs, n (%)	**…**	**…**	**…**
*Chlamydia trachomatis* infection	34 (31.2)	19 (33.9)	15 (28.3)
*Neisseria gonorrhoeae* infection	15 (13.8)	5 (8.9)	10 (18.9)
*Mycoplasma genitalium* infection	7 (6.4)	3 (5.4)	4 (7.5)
Symptoms of STIs within 6 m, n (%)	69 (63.3)	35 (62.5)	34 (64.2)
Previous STIs within 1 y, n (%)	80 (73.4)	36 (64.3)	44 (83.0)
Substance use, n (%)	49 (45.0)	30 (53.6)	19 (35.9)
Anal-penile sex, n (%)	88 (82.2)	47 (87.0)	41 (77.4)
Oral-penile sex, n (%)	78 (71.6)	38 (67.9)	40 (75.5)
Oral-anal sex, n (%)	31 (28.4)	16 (28.6)	15 (28.3)
Having a partner known to have STI, n (%)	28 (25.7)	10 (17.9)	18 (34.0)
Number of sex partners >5 within 3 m, n (%)	13 (11.9)	4 (7.1)	9 (17.0)
Inconsistent condom use, n (%)	85 (78.0)	47 (83.9)	38 (71.7)
Use of mobile dating application, n (%)	71 (65.1)	39 (69.6)	32 (60.4)

Abbreviations: ART, antiretroviral therapy; BPG, benzathine penicillin G; CRO, ceftriaxone; Doxy, doxycycline; HBsAg, hepatitis B virus surface antigen; HCV, hepatitis C virus; IQR, interquartile range; RPR, rapid plasma reagin; STI, sexually transmitted infection.

^a^Participants testing positive for anti-HCV IgG and/or HCV RNA in the past 12 m.

^b^Of the 109 participants, 39 had *T. pallidum* detected at one or more sampling sites, whereas 70 had no *T. pallidum* detected at any sampling site.

**Figure 2.**
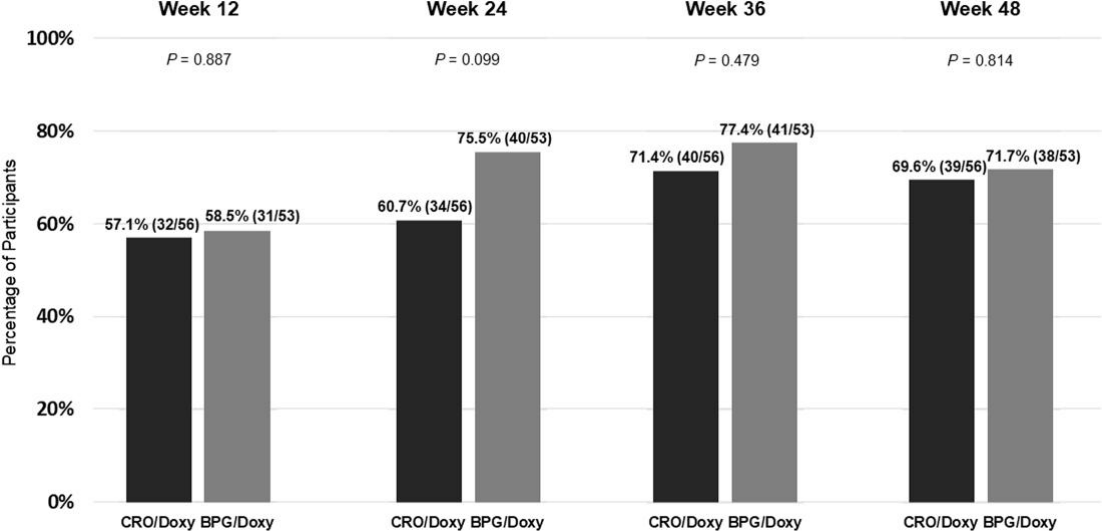
Serologic responses of syphilis in the ITT population at 12, 24, 36, and 48 wks. Legend: black bar, CRO/Doxy group; gray bar, BPG/Doxy group. Abbreviation: ITT, intention-to-treat; BPG, benzathine penicillin G; CRO, ceftriaxone; Doxy, doxycycline.

**Figure 3.**
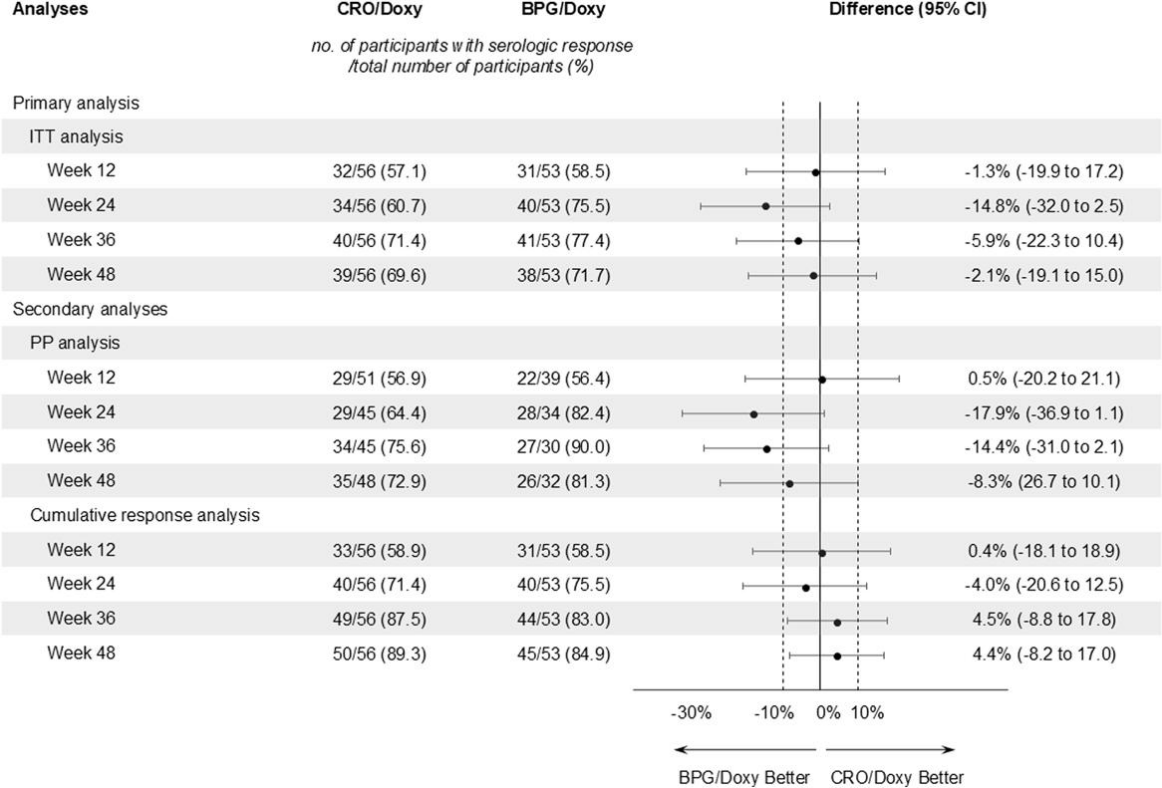
Primary and secondary analyses of serologic responses of syphilis at 12, 24, 36, and 48 wks. Legend: dots, the risk difference between the CRO/Doxy and BPG/Doxy groups; bars, the 95% CI of the risk difference between the CRO/Doxy and BPG/Doxy groups. Abbreviation: ITT, intention-to-treat; PP, per-protocol; CI, confidence interval; BPG, benzathine penicillin G; CRO, ceftriaxone; Doxy, doxycycline.

The serologic responses of syphilis are depicted in Figures 2 and 3. In the ITT analysis that included all participants, compared to PWH receiving BPG/Doxy, those treated with CRO/Doxy demonstrated a numerically lower serologic response rate at 24 weeks (60.7% vs 75.5%; difference, −14.8 percentage points; 95% CI, −32.0 to 2.5) (Figures 2 and 3). In the PP analysis, which excluded participants with a baseline co-infection requiring additional antibiotics active against *T. pallidum* or those who received such antibiotics during follow-up, participants treated with CRO/Doxy showed a numerically lower serologic response rate compared to those treated with BPG/Doxy at 24 weeks (64.4% vs 82.4%; difference, −17.9 percentage points; 95% CI, −36.9 to 1.1) (Figure 3).

Time to cumulative serologic response is depicted in Supplementary Figure 1. There was no significant difference in the cumulative serologic response between participants receiving CRO/Doxy and those receiving BPG/Doxy (Supplementary Figure 1A, Log-rank test, *P* = .855; hazard ratio [HR], 1.04; 95% CI, .69–1.55).

Reasons for treatment failure in the ITT analysis are demonstrated in Supplementary Figure 2. The most common reason for treatment failure at 24 weeks was the absence of a ≥ 4-fold decline in RPR titers. This occurred in 26.8% (15/56) of participants receiving CRO/Doxy and in 15.1% (8/53) of those receiving BPG/Doxy. The evolutions of treatment response are demonstrated in Supplementary Figure 3. Among the participants receiving CRO/Doxy who achieved serologic response after treatment, at the next follow-up timepoint, the proportion of participants with either a RPR titer within one dilution or a ≥ 4-fold increase of baseline was 10.0% (1/10) at week 4, 9.4% (3/32) at week 12, 5.9% (2/34) at week 24, and 10.0% (4/40) at week 36. In contrast, among the participants receiving BPG/Doxy who achieved a serologic response, the proportions with such a titer change were 0% at week 4 and 12, 2.5% (1/40) at week 24, and 4.9% (2/41) at week 36.

At 4 weeks, the microbiologic response of *T. pallidum* among the participants receiving CRO/Doxy (17/18 [94.4%]) was not statistically different from that among those receiving BPG/Doxy (20/21 [95.2%]) (Table 2). The microbiologic responses of *C. trachomatis* and *M. genitalium* were not statistically different between CRO/Doxy and BPG/Doxy groups (16/19 [84.2%] vs 13/15 [86.7%] for *C. trachomatis*, and 0/3 [0%] vs 2/4 [50.0%] for *M. genitalium*).

**Table 2.** Microbiologic Responses of *Treponema pallidum*, *Chlamydia trachomatis*, and *Mycoplasma genitalium* at 4 Weeks

	CRO/Doxy Group	BPG/Doxy Group	*P*
*T. pallidum*	94.4% (17/18)	95.2% (20/21)	>.999
*C. trachomatis*	84.2% (16/19)	86.7% (13/15)	>.999
*M. genitalium*	0.0% (0/3)	50.0% (2/4)	.429

At week 4, microbiologic response of syphilis was defined as a *T. pallidum* PCR cycle threshold (Ct) value >38 at all sampling sites, including oral rinse, rectal swab, and urethral swab, using an in-house PCR assay to amplify the 47 kDa antigen gene. Microbiologic response of *C. trachomatis* and *M. genitalium* at week 4 were defined as negative PCR results at all sampling sites using a multiplex real-time PCR assay (Allplex STI Essential Assay; Seegene Inc, Seoul, Republic of Korea).

Abbreviations: BPG, benzathine penicillin G; CRO, ceftriaxone; Doxy, doxycycline.

The geometric mean titers (GMT) of the participants receiving CRO/Doxy or BPG/Doxy with and without concomitant gonorrhea at baseline are depicted in Supplementary Figure 4 and Supplementary Table 1. Participants receiving BPG/Doxy showed a lower GMT compared to those receiving CRO/Doxy at weeks 4, 12, 24, 36, and 48. Among the participants receiving BPG/Doxy, those with concomitant gonorrhea at baseline and subsequently received additional CRO had numerically lower GMTs than those without concomitant gonorrhea at weeks 4, 12, 24, 36, and 48. The baseline characteristics of the participants receiving BPG/Doxy without concomitant gonorrhea were generally similar to those receiving CRO/Doxy (Supplementary Table 2). In the univariable analysis, participants who received additional CRO for baseline concomitant gonorrhea showed a non-significant trend toward a higher serologic response at week 24 (OR, 2.00; 95% CI, .40–9.91) but not at week 48 (OR, .53; 95% CI, .14–2.04) (Supplementary Tables 3 and 4).

Adverse events are listed in Supplementary Table 5. Compared to the participants receiving BPG/Doxy, those receiving CRO/Doxy demonstrated significantly more injection site pain or swelling (24/56 [42.9%] vs 11/53 [20.8%], *P* = .014) and Jarisch-Herxheimer reaction (10/56 [17.9%] vs 2/53 [3.8%], *P* = .029). A higher incidence of injection site pain was observed after 38 participants in the CRO/Doxy group were treated. Consequently, for the remaining participants, CRO was diluted with lidocaine. Following this adjustment, the incidence of injection site pain was comparable to that in the BPG/Doxy group (4/18 [22.2%] vs 11/53 [20.8%], *P* > .999).

## DISCUSSION

In this study comparing CRO/Doxy versus BPG/Doxy for the treatment of early syphilis in PWH, we found that CRO/Doxy did not show noninferiority in terms of serologic response compared to BPG/Doxy at 24 weeks in the ITT population. However, the microbiologic responses of syphilis, chlamydia, and *M. genitalium* infection were comparable for the two groups at week 4. Treatment with CRO/Doxy showed more injection site pain or swelling and Jarisch-Herxheimer reaction compared to treatment with BPG/Doxy.

The serologic response rates in our ITT population were lower than those reported in other studies for early syphilis. Several factors may account for this difference. First, our study population was unique in exhibiting particularly high-risk sexual behaviors: 85.3% of the participants had had a history of syphilis, 73.4% reported previous STIs within the past year, and 78.0% used condoms inconsistently (Table 1). This likely contributed to an increased risk of syphilis reinfection. A high rate of STI co-infection was observed in our study, with 31.2% with chlamydia and 13.8% with gonorrhea at baseline. In contrast, the study by Ubals et al., which reported a 100% serologic response rate at 12 months among participants receiving BPG, had only 11.9% with chlamydia and 4.8% with gonorrhea at baseline [21]. Second, some studies did not classify relapse or reinfection as treatment failures [16, 22, 23]. For instance, the study by Reidner et al. censored participants once they achieved a serologic response [22]. Third, our study included participants who were lost to follow-up and classified as treatment failures, whereas some previous randomized trials excluded them from the primary analysis [16, 21, 23]. For example, the study by Stafylis et al., which reported a 90% serologic response rate in PWH receiving BPG, excluded 48.3% (28/58) of participants from the ITT population due to withdrawal or missed follow-up visits [23]. Most observational studies also did not classify participants who were lost to follow-up as treatment failures, which may have led to an overestimation of serologic response rates [18, 24–26]. Fourth, our study exclusively enrolled PWH, whereas other studies included both PWH and HIV-negative individuals [16, 21, 22, 27]. This aligns with previous findings showing that PWH with syphilis tend to have lower serologic response rates compared to HIV-negative individuals [26]. Last, the serologic response rates in the CRO/Doxy group in our ITT analysis were lower than those reported in the randomized trial by Cao et al. (90.2% at 6 months) [16]. The study administered 1.0 g of CRO daily for 10 days. To date, no other studies have evaluated the efficacy 1.0 g CRO administered for fewer than 7 days for early syphilis [13–16]. Therefore, it is not unexpected that our study, which used a single-dose CRO regimen, yielded a lower response rate compared to prior trials.

The design of our study did not exclude participants with baseline co-infections, which was a scenario likely common in real-world clinical settings. However, this design introduced a potential issue: 10 participants in the BPG/Doxy group were found to have gonorrhea and an additional dose of CRO was administered, which may have inadvertently increased the serologic response rate for syphilis in the BPG/Doxy group. We addressed this issue by performing a PP analysis, which excluded participants with a baseline co-infection or those who received additional antibiotics during follow-up. This approach provides a cleaner assessment of the true pharmacologic effect of the study drugs. However, this may also have selected a subgroup within the BPG/Doxy group with lower risk behaviors.

At week 4, the CRO/Doxy group demonstrated a similar microbiologic response rate to the BPG/Doxy group. The higher rate of Jarisch-Herxheimer reaction observed in participants receiving CRO/Doxy may suggest that this regimen elicits a robust bactericidal effect. In the cumulative serologic response analysis, where relapse or reinfection is not accounted for, participants receiving CRO/Doxy showed numerically higher serologic response rates at weeks 36 and 48 compared to those receiving BPG/Doxy. These findings support that a single dose of CRO may exhibit good early-phase bactericidal activity but raise concerns about whether a single dose of CRO is sufficient to maintain long-term therapeutic efficacy for killing *T. pallidum*. A general principle in the treatment of early syphilis is to maintain antibiotic concentrations above the inhibitory threshold for 7 to 10 days [28, 29]. Given that the half-life of a single-dose CRO is around 8 hours, a single-dose CRO is not likely to provide additional therapeutic coverage to the short course of doxycycline. While *T. pallidum* replicates approximately every 30 to 33 hours, it replicates more slowly in late syphilis [30]. Under such circumstances, the difference in duration above bactericidal concentrations between CRO and BPG is more clinically significant. *T. pallidum* may sequester in the central nervous system or the eye. In these cases, CRO may show more favorable CSF penetration compared to BPG [31]. However, given the slow replication rate of *T. pallidum*, a single dose of CRO may be insufficient for eradication.

In the present study, we add Doxy to both regimens to expand antimicrobial coverage to chlamydia. We did not compare CRO/Doxy to BPG alone, which was the standard of care for early syphilis. CRO/Doxy have the advantage of treating coinfections; however, given the shorter half-life of CRO, CRO/Doxy was probably unlikely to show better efficacy for early syphilis than BPG alone. Extending doxycycline from 7 to 14 days in combination with a single-dose CRO might improve its efficacy.

Our study has several limitations. First, the sample size calculation was based on a prior study with different dosing and analytical strategies, which may have led to an overestimation of the efficacy with single-dose CRO and, consequently, affected our sample size estimation. The small sample size limited the precision of estimation. Second, the open-label design may have introduced bias, although the treatment response was based on changes of RPR titer. Third, the absence of whole-genome sequencing limited our ability to distinguish treatment failure from reinfection. *T. pallidum* PCR was performed only on non-lesion samples, which has a lower detection rate than lesion samples. Fourth, routine CSF examinations were not performed, potentially missing cases of asymptomatic neurosyphilis. Fifth, adverse events were self-reported, raising the possibility of reporting bias. Sixth, the study did not account for the potential effect of doxycycline post-exposure prophylaxis (Doxy-PEP), although Doxy-PEP was not widely adopted at the start of the trial. Lastly, the findings are limited to male PWH and may not be generalizable to broader populations.

In conclusion, treatment with a single dose of CRO combined with a 7-day course of doxycycline did not demonstrate non-inferiority compared to that with a single dose of BPG combined with a 7-day course of doxycycline among PWH with early syphilis over a 24-week follow-up period. Our study suggests that a single-dose CRO in combination with a 7-day course of doxycycline should not be used for the treatment of early syphilis.

## Supplementary Material

ofag483_Supplementary_Data

## Supplementary Data

Supplementary materials are available at *Open Forum Infectious Diseases* online. Consisting of data provided by the authors to benefit the reader, the posted materials are not copyedited and are the sole responsibility of the authors, so questions or comments should be addressed to the corresponding author.
