# Supplementary material for: Single-dose Ceftriaxone Plus 7-Day Doxycycline Versus Single-dose Benzathine Penicillin G Plus 7-Day Doxycycline in the Treatment of Early Syphilis in People With HIV: a Pilot Randomized Noninferiority Trial
Source: Open Forum Infect Dis. 2026 Aug 8;13(8):ofag483. doi: 10.1093/ofid/ofag483 (PMC13481986; doi:10.1093/ofid/ofag483)
Supplement: Supplementary file 1 [file ofag483_supplementary_data.docx]

**Supplementary appendix**

Supplementary materials to: *Single-dose ceftriaxone plus 7-day doxycycline versus single-dose benzathine penicillin G plus 7-day doxycycline in the treatment of early syphilis in people with HIV: a pilot randomized noninferiority trial*

**Table of Contents**

p. 2	Table S1. GMT of RPR titers and the natural logarithm of GMT of RPR titers in the ITT population

p. 4	Table S2. Baseline characteristics of participants in the CRO/Doxy group and those in the BPG/Doxy group without concurrent *N. gonorrhoeae* infection

p. 6	Table S3. Factors associated with serologic response at week 24 in the ITT population

p. 8	Table S4. Factors associated with serologic response at week 48 in the ITT population

p. 10	Table S5. Treatment-emergent adverse effects for the two groups

p. 11	Figure S1. Time to cumulative serologic response in the ITT population

p. 13	Figure S2. Reasons for treatment failure in the ITT population

p. 14	Figure S3. Evolution of treatment responses in the ITT population

p. 16	Figure S4. GMT of RPR titers in the ITT population

**Table S1. GMT of RPR titers and the natural logarithm of GMT of RPR titers in the ITT population**

|  | **Baseline** | **4 weeks** | **12 weeks** | **24 weeks** | **36 weeks** | **48 weeks** |
| --- | --- | --- | --- | --- | --- | --- |
|  | **GMT of RPR titers (95% CI)** | | | | | |
| CRO/Doxy group (n=56) | 107.63 (74.60, 155.29) | 73.13 (49.34, 108.39) | 35.53 (25.39, 49.72) | 20.35 (14.26, 29.04) | 13.60 (9.15, 20.22) | 10.37 (7.36, 14.63) |
| BPG/Doxy group (n=53) | 93.54 (66.14, 146.81) | 62.10 (39.08, 98.69) | 22.93 (15.82, 33.25) | 10.99 (7.33, 16.47) | 8.42 (5.43, 13.05) | 5.96 (3.62, 9.82) |
| BPG/Doxy group without concomitant gonorrhea at baseline (n=43) | 92.72 (60.16, 142.91) | 64.00 (36.92, 110.94) | 24.57 (15.83, 38.14) | 11.95 (7.45, 19.17) | 9.51 (5.62, 16.12) | 6.39 (3.77, 10.84) |
| BPG/Doxy group with concomitant gonorrhea at baseline (n=10) | 128.00 (38.87, 421.53) | 54.86 (21.99, 136.88) | 17.15 (8.69, 33.83) | 8.00 (3.34, 19.18) | 5.44 (2.44, 12.16) | 4.00 (0.54, 29.71) |
|  | **Natural logarithm of the GMT of RPR titers (95% CI)** | | | | | |
| CRO/Doxy group (n=56) | 4.68 (4.31, 5.04) | 4.29 (3.89, 4.69) | 3.57 (3.23, 3.91) | 3.01 (2.66, 3.37) | 2.61 (2.21, 3.01) | 2.34 (2.00, 2.68) |
| BPG/Doxy group (n=53) | 4.59 (4.19, 4.99) | 4.13 (3.67, 4.59) | 3.13 (2.76, 3.50) | 2.40 (1.99, 2.80) | 2.13 (1.69, 2.57) | 1.78 (1.29, 2.28) |
| BPG/Doxy group without concomitant gonorrhea at baseline (n=43) | 4.53 (4.10, 4.96) | 4.16 (3.61, 4.71) | 3.20 (2.76, 3.64) | 2.48 (2.01, 2.95) | 2.25 (1.73, 2.78) | 1.86 (1.33, 2.38) |
| BPG/Doxy group with concomitant gonorrhea at baseline (n=10) | 4.85 (3.66, 6.04) | 4.00 (3.09, 4.92) | 2.84 (2.16, 3.52) | 2.08 (1.20, 2.95) | 1.69 (0.89, 2.50) | 1.39 (-0.62, 3.39) |
| P value^a^ | 0.744 | 0.588 | 0.082 | 0.024 | 0.104 | 0.061 |
| P value^b^ | 0.768 | 0.831 | 0.162 | 0.054 | 0.151 | 0.131 |

**Abbreviations**: GMT, geometric mean titer; RPR, rapid plasma reagin; ITT, intention-to-treat; CI, confidence interval

^a^The P value between CRO/Doxy group and BPG/Doxy group is calculated by t-test.

^b^The P values between CRO/Doxy group, BPG/Doxy group without concomitant gonorrhea at baseline, and BPG/Doxy group with concomitant gonorrhea at baseline are calculated by ANOVA test.

**Footnote:** The GMT of RPR titers is calculated by averaging the natural logarithms of the titers, and then exponentiating the result.

**Table S2. Baseline characteristics of the participants in the CRO/Doxy group and those in the BPG/Doxy group without concurrent *N. gonorrhoeae* infection**

| **Baseline Characteristics** | **CRO/Doxy group (N=56)** | **BPG/Doxy group without concurrent *N. gonorrhoeae* infection at baseline (N=43)** |
| --- | --- | --- |
| Male, n (%) | 56 (100) | 43 (100) |
| Age, median (IQR), years | 38 (33-46) | 42 (35-48) |
| Receiving ART at baseline, n (%) | 56 (100) | 42 (97.7) |
| CD4 count at baseline, median (IQR), cells/mm^3^ | 556 (477-794) | 554 (422-699) |
| Plasma HIV RNA load <200 copies/mL, n (%) | 51 (91.1) | 36 (83.7) |
| HBsAg positivity, n (%) | 7 (12.5) | 2 (4.7) |
| HCV infection^a^, n (%) | 13 (23.2) | 9 (20.9) |
| History of previous syphilis, n (%) | 49 (87.5) | 35 (81.4) |
| Syphilis stage, n (%) |  |  |
| Primary | 3 (5.4) | 3 (7.0) |
| Secondary | 22 (39.3) | 13 (30.2) |
| Early latent | 31 (55.4) | 27 (62.8) |
| RPR titer, median (IQR) | 128 (64-256) | 128 (64-256) |
| RPR titer ≤ 1:32, n (%) | 12 (21.4) | 10 (23.3) |
| RPR titer ≥ 1:64, n (%) | 44 (78.6) | 33 (76.7) |
| *T. pallidum* DNA detection, n (%) | 18 (32.1) | 17 (39.5) |
| Interval between RPR testing to treatment administration, median (IQR), days | 5 (0-9) | 4 (0-8) |
| Concurrent bacterial STIs, n (%) |  |  |
| *Chlamydia trachomatis* infection | 19 (33.9) | 9 (20.9) |
| *Neisseria gonorrhoeae* infection | 5 (8.9) | 0 (0.0) |
| *Mycoplasma genitalium* infection | 3 (5.4) | 2 (4.7) |
| Symptoms of STIs within 6 months, n (%) | 35 (62.5) | 27 (62.8) |
| Previous STIs within 1 year, n (%) | 36 (64.3) | 35 (81.4) |
| Substance use, n (%) | 30 (53.6) | 13 (30.2) |
| Anal-penile sex, n (%) | 47 (87.0) | 32 (74.4) |
| Oral-penile sex, n (%) | 38 (67.9) | 32 (74.4) |
| Oral-anal sex, n (%) | 16 (28.6) | 11 (25.6) |
| Having a partner known to have STI, n (%) | 10 (17.9) | 12 (27.9) |
| Number of sex partners >5 within 3 months, n (%) | 4 (7.1) | 9 (20.9) |
| Inconsistent condom use, n (%) | 47 (83.9) | 35 (81.4) |
| Use of mobile dating application, n (%) | 39 (69.6) | 25 (58.1) |

**Abbreviations:** ART, antiretroviral therapy; BPG, benzathine penicillin G; CRO, ceftriaxone; Doxy, doxycycline; HBsAg, hepatitis B virus surface antigen; HCV, hepatitis C virus; IQR, interquartile range; RPR, rapid plasma reagin; STI, sexually transmitted infection.

^a^Participants testing positive for anti-HCV IgG and/or HCV RNA in the past 12 months.

**Table S3. Factors associated with serologic response at week 24 in the ITT population**

|  | **Univariable analysis** | | **Multivariable analysis** | |
| --- | --- | --- | --- | --- |
|  | **OR (95% CI)** | ***P*** | **AOR (95% CI)** | ***P*** |
| Study drug received at baseline |  |  |  |  |
| BPG/Doxy | - | - |  |  |
| CRO/Doxy | 0.50 (0.22-1.15) | 0.101 |  |  |
| Additional antibiotics active against *T. pallidum* up to 24 weeks |  |  |  |  |
| For baseline concurrent gonorrhea | 2.00 (0.40-9.95) | 0.397 |  |  |
| For other reasons | 2.31 (0.71-7.48) | 0.162 |  |  |
| Age, per 1-year increase | 0.98 (0.94-1.02) | 0.366 |  |  |
| Receiving ART at baseline | -^a^ | -^a^ |  |  |
| CD4 count at baseline, per 10-cells/mm^3^ increase | 1.00 (0.98-1.02) | 0.876 |  |  |
| Plasma HIV RNA load at screening, per 1-log_10_ copies/mL increase | 0.64 (0.35-1.15) | 0.133 |  |  |
| HBsAg positivity | 0.56 (0.14-2.24) | 0.413 |  |  |
| HCV infection^b^ | 0.86 (0.32-2.27) | 0.757 |  |  |
| Syphilis stage |  |  |  |  |
| Primary or secondary | - | - |  |  |
| Early latent | 0.67 (0.43-1.03) | 0.067 | 0.87 (0.54-1.41) | 0.576 |
| RPR titer at baseline |  |  |  |  |
| RPR titer ≤ 1:32 | - | - |  |  |
| RPR titer ≥ 1:64 | 4.27 (1.65-11.03) | 0.003 | 2.75 (0.99-7.66) | 0.053 |
| *T. pallidum* DNA detection at baseline | 5.10 (1.78-14.59) | 0.002 | 3.71 (1.23-11.13) | 0.020 |
| Symptoms of STIs within 6 months | 1.75 (0.77-4.00) | 0.181 |  |  |
| Previous STIs within 1 year | 0.55 (0.17-1.82) | 0.330 |  |  |
| Substance use | 0.96 (0.43-2.14) | 0.913 |  |  |
| Having a partner known to have STI | 0.65 (0.27-1.59) | 0.347 |  |  |
| Number of sex partners >5 within 3 months | 2.88 (0.60-13.77) | 0.185 |  |  |
| Inconsistent condom use | 0.83 (0.24-2.84) | 0.762 |  |  |
| Use of mobile dating application | 0.96 (0.41-2.24) | 0.931 |  |  |

**Abbreviations:** ART, antiretroviral therapy; BPG, benzathine penicillin G; CRO, ceftriaxone; Doxy, doxycycline; HBsAg, hepatitis B virus surface antigen; HCV, hepatitis C virus; ITT, intention-to-treat; RPR, rapid plasma reagin; STI, sexually transmitted infection.

^a^All participants who achieved a serologic response at 24 weeks had received ART at baseline.

^b^Participants testing positive for anti-HCV IgG and/or HCV RNA in the past 12 months.

**Table S4. Factors associated with serologic response at week 48 in the ITT population**

|  | **Univariable analysis** | | **Multivariable analysis** | |
| --- | --- | --- | --- | --- |
|  | **OR (95% CI)** | ***P*** | **AOR (95% CI)** | ***P*** |
| Study drug received at baseline |  |  |  |  |
| BPG/Doxy | - | - |  |  |
| CRO/Doxy | 0.90 (0.39-2.08) | 0.801 |  |  |
| Additional antibiotics active against *T. pallidum* up to 48 weeks |  |  |  |  |
| For baseline concurrent gonorrhea | 0.53 (0.14-2.04) | 0.360 |  |  |
| For other reasons | 1.65 (0.50-5.41) | 0.408 |  |  |
| Age, per 1-year increase | 1.02 (0.97-1.07) | 0.460 |  |  |
| Receiving ART at baseline | -^a^ | -^a^ |  |  |
| CD4 count at baseline, per 10-cells/mm^3^ increase | 0.99 (0.97-1.01) | 0.275 |  |  |
| Plasma HIV RNA load at screening, per 1-log_10_ copies/mL increase | 0.51 (0.27-0.98) | 0.044 | 0.41 (0.18-0.93) | 0.033 |
| HBsAg positivity | 1.36 (0.27-6.95) | 0.711 |  |  |
| HCV infection^b^ | 0.83 (0.30-2.29) | 0.725 |  |  |
| Syphilis stage |  |  |  |  |
| Primary or secondary | - | - |  |  |
| Early latent | 0.79 (0.51-1.23) | 0.301 |  |  |
| RPR titer at baseline |  |  |  |  |
| RPR titer ≤ 1:32 | - | - |  |  |
| RPR titer ≥ 1:64 | 4.73 (1.80-12.38) | 0.002 | 4.20 (1.46-12.10) | 0.008 |
| *T. pallidum* DNA detection at baseline | 3.78 (1.31-10.89) | 0.014 | 2.86 (0.91-9.00) | 0.072 |
| Symptoms of STIs within 6 months | 1.21 (0.51-2.88) | 0.660 |  |  |
| Previous STIs within 1 year | 0.28 (0.06-1.31) | 0.106 |  |  |
| Substance use | 0.63 (0.27-1.46) | 0.280 |  |  |
| Having a partner known to have STI | 0.93 (0.36-2.42) | 0.885 |  |  |
| Number of sex partners >5 within 3 months | 1.30 (0.33-5.11) | 0.703 |  |  |
| Inconsistent condom use | 0.69 (0.18-2.66) | 0.586 |  |  |
| Use of mobile dating application | 1.36 (0.57-3.24) | 0.489 |  |  |

**Abbreviations:** ART, antiretroviral therapy; BPG, benzathine penicillin G; CRO, ceftriaxone; Doxy, doxycycline; HBsAg, hepatitis B virus surface antigen; HCV, hepatitis C virus; ITT, intention-to-treat; RPR, rapid plasma reagin; STI, sexually transmitted infection.

^a^All participants who achieved a serologic response at 48 weeks had received ART at baseline.

^b^Participants testing positive for anti-HCV IgG and/or HCV RNA in the past 12 months.

**Table S5. Treatment-emergent adverse effects for the two groups**

|  | **CRO/Doxy group (n=56)** | **BPG/Doxy group (n=53)** | **P** |
| --- | --- | --- | --- |
| Any adverse events | 26 (46.4) | 15 (28.3) | 0.051 |
| Injection site pain or local swelling | 24/56 (42.9) | 11/53 (20.8) | 0.014 |
| Not diluted with lidocaine | 20/38 (52.6) | 11/53 (20.8) | 0.002^a^ |
| Diluted with lidocaine | 4/18 (22.2) | - | >0.999^b^ |
| Jarisch-Herxheimer reaction | 10 (17.9) | 2 (3.8) | 0.029 |
| Diarrhea | 2 (3.6) | 1 (1.9) | >0.999 |
| Myalgia | 0 (0.0) | 1 (1.9) | >0.999 |
| Back pain | 1 (1.8) | 0 (0.0) | >0.999 |
| Mouth swelling | 1 (1.8) | 0 (0.0) | >0.999 |
| Throat itchiness | 0 (0.0) | 1 (0.0) | >0.999 |
| Acne | 0 (0.0) | 1 (0.0) | >0.999 |

**Abbreviations:** BPG, benzathine penicillin G; CRO, ceftriaxone; Doxy, doxycycline

^a^Comparing CRO/Doxy group not diluted with lidocaine (n=38) to BPG/Doxy group (n=53).

^b^Comparing CRO/Doxy group diluted with lidocaine (n=18) to BPG/Doxy group (n=53).

**Figure S1. Time to cumulative serologic response in the ITT population**

**Abbreviations:** ITT, intention-to-treat; CRO, ceftriaxone; Doxy, doxycycline; BPG, benzathine penicillin G; HR, hazard ratio; CI, confidence interval; PS, primary syphilis; SS, secondary syphilis; ELS, early latent syphilis; RPR, rapid plasma reagin; TP-DNA, *T. pallidum* DNA

**Footnote:** (A) The log-rank test and hazard ratio for CRO/Doxy group compared with BPG/Doxy group; (B) age <40 years vs ≥40 years; (C) The log-rank test and hazard ratio for early latent syphilis compared with primary or secondary syphilis; (D) The log-rank test and hazard ratio for RPR titer ≥1:64 compared with RPR titer ≤1:32; (E) The log-rank test and hazard ratio for TP-DNA detection compared with no TP-DNA detection at baseline; (F) The log-rank test and hazard ratio for TP-DNA detection compared with no TP-DNA detection at baseline, in participants with RPR titer ≥1:64 at baseline; (G) The log-rank test and hazard ratio for CRO/Doxy group compared with BPG/Doxy group, in participants with TP-DNA detection at baseline; (H) The log-rank test and hazard ratio for CRO/Doxy group compared with BPG/Doxy group, in participants without TP-DNA detection at baseline. Participants with TP-DNA detected at baseline demonstrated a higher cumulative serologic response rate (Panel E), and this effect persisted among those with high baseline RPR titers (Panel F). This is similar to the findings of a previous observational study, where syphilis cases with detected TP-DNA at baseline had a higher rate of serologic response, regardless of clinical stages and RPR titers.^1^

**Reference:**

1. Wu TY, Lin KY, Sun HY, et al. Detection of *Treponema pallidum* DNA for diagnosis, resistance identification, and treatment outcome prediction in early syphilis among men who have sex with men. *Clin Microbiol Infect* 2025; **31**(6): 1026-32.

**Figure S2. Reasons for treatment failure in the ITT population**

**Abbreviation:** ITT, intention-to-treat; RPR, rapid plasma reagin; BPG, benzathine penicillin G; CRO, ceftriaxone; Doxy, doxycycline

**Legend:** broad diagonal stripes, ≥4-fold increase in RPR titers; thin diagonal stripes, receipt of BPG following a ≥4-fold increase in RPR titers; white dots, lack of ≥4-fold decline in RPR titers; horizontal stripes, receipt of BPG following a lack of ≥4-fold decline in RPR titers; grey dots, missed study follow-up

**Figure S3. Evolution of treatment responses in the ITT population**

**Abbreviations:** ITT, intention-to-treat; CRO, ceftriaxone; Doxy, doxycycline; BPG, benzathine penicillin G

**Legend:** blue, ≥4-fold decline in RPR titers compared to baseline; orange, within one dilution in RPR titers compared to baseline; red, ≥4-fold increase in RPR titers compared to baseline; green, receipt of BPG; cyan, missed study follow-up; numbers in the middle of the bars indicate the number of participants in each category at the specific follow-up timepoints; small italic numbers on the right side of the bars indicate the number of participants in each category who transitioned to a new category at the next follow-up timepoint

**Footnote:** (A) CRO/Doxy group. (B) BPG/Doxy group.

**Figure S4. GMT of RPR titers in the ITT population**

**Abbreviations:** GMT, geometric mean titer; RPR, rapid plasma reagin; ITT, intention-to-treat; CRO, ceftriaxone; Doxy, doxycycline; BPG, benzathine penicillin G

**Legend:** blue, CRO/Doxy group; dark green, BPG/Doxy group; light green, BPG/Doxy group without concomitant gonorrhea at baseline; orange, BPG/Doxy group with concomitant gonorrhea at baseline

**Footnote:** (A) The GMT of RPR titers among CRO/Doxy group and BPG/Doxy group. (B) The GMT of RPR titers among CRO/Doxy group, and BPG/Doxy group with and without concomitant gonorrhea at baseline. The GMT of RPR titers is calculated by averaging the natural logarithms of the titers and then exponentiating the result.
